# Pain medication and nerve injuries in upper or lower limb—a Swedish national registry study

**DOI:** 10.1097/PR9.0000000000001391

**Published:** 2026-01-16

**Authors:** Lars B. Dahlin, Raquel Perez, Malin Zimmerman, Erika Nyman, Emma Dahlin, Drifa Frostadottir, Juan Merlo

**Affiliations:** a Department of Translational Medicine—Hand Surgery, Lund University, Skåne University Hospital, Malmö, Sweden; b Department of Biomedical and Clinical Sciences, Linköping University, Linköping, Sweden; c Department of Hand Surgery, Skåne University Hospital, Malmö, Sweden; d Unit for Social Epidemiology, Department of Clinical Sciences (Malmö), Faculty of Medicine, Lund University, Malmö, Sweden; e Department of Orthopedics, Helsingborg Hospital, Helsingborg, Sweden; f Clinical Department of Hand Surgery, Plastic Surgery and Burns, Linköping University Hospital, Linköping, Sweden; g Center for Primary Health Research, Region Skåne, Malmö, Sweden

**Keywords:** Peripheral nerve injury, Opioids, Upper limb, Lower limb, Pain, Socioeconomic factors

## Abstract

**Introduction:**

Severe symptoms and disability may remain after upper and lower limb nerve injuries with an additional risk of persistent pain and consumption of pain medication.

**Objectives:**

To examine whether upper and lower limb nerve injuries, potentially causes of pain, are associated with risk of using pain medication.

**Methods:**

Drug use at an individual level was analysed in a large Swedish record-linkage database covering 6,579,612 individuals (age ≥18 years; first-ever nonsurgically [n = 5,249] and surgically treated [n = 3,669] upper limb and nonsurgically [n = 1,260] and surgically treated [n = 14] lower limb nerve injuries; International Classification of Diseases, 10th version and NOMESCO codes; 90-day washout period after surgery). Associations were expressed as relative risks (95% confidence interval), unadjusted and adjusted for demographic and socioeconomic factors.

**Results:**

Adjusted risk for use of pain medications during the first year after diagnosis and/or surgery was higher in upper (4.85 [4.51–5.23]) and lower (5.60 [4.85–6.48]) limb nerve injuries than in the general population and was considerably lower if surgery was performed (0.31 [0.26–0.36]). Risk during the second year declined but remained. Consumption of pain medication during the first year increased risk of use during the second year (11.45 [11.35–11.56]). Older age, female sex, living alone, low-middle income, and immigrant status were associated with a higher risk.

**Conclusion:**

Upper and lower limb nerve injuries substantially increase the risk of long-term use of pain medication. Surgical treatment reduces the risk during the first year, indicating the relevance of appropriate surgical treatment, but demographic and socioeconomic disparities remain essential determinants. Notably, previous consumption during the first year increases the risk of consumption in the second year. Despite being a third-line therapy for neuropathic pain, opioids accounted for many prescriptions.

## Abstract 2

Supplemental Digital Content is Available in the Text.

Although individuals with upper or lower limb nerve injuries, treated either nonsurgically or surgically, face a higher risk of using pain medication, surgical treatment reduces that risk.

## 1. Introduction

Outcome regarding relatively common traumatic nerve injuries in upper and lower limbs^28,34^ must be carefully evaluated.^23^ Severe symptoms and disability^13,14,42^ may persist despite surgery,^6,14,36,42^ particularly after major nerve trunk injuries.^10,13,14,32,42^ Neuropathic pain is difficult to treat and occurs despite surgery. Half of patients report persistent pain long after upper limb surgery, of which 73% is defined as neuropathic pain.^32^ Pain and cold sensitivity affect activities of daily living,^13,14,27,31,32^ with findings differing between the sexes.^33^ Less is reported about lower limb nerve injuries.^1,28^ There is a lack of knowledge and evidence-based stepwise guidelines concerning the efficacy of treatment strategies for pain after peripheral nerve injuries.^11^

Opioids, extensively used to treat pain despite an insufficient response, cause substantial residual side effects with addiction.^3,29^ Despite the existence of protocols for standardized postoperative opioid prescription, variations in adherence occur after surgery for ulnar nerve entrapment (UNE).^15^ Preoperative misuse of opioids in individuals scheduled for orthopaedic surgery may induce increased morbidity and even mortality.^30^ A nerve injury is also reported to induce receptor alterations.^26,37,41^ Crucially, the ongoing opioid pandemic necessitates understanding function, expression, pharmacology, and regulation of endogenous opioid systems in pain,^18^ different experimental models,^44^ risk of opioid consumption in upper and lower limb nerve injuries, irrespective of surgery, and associations with socioeconomic factors and other disorders.^17,40,43^ Nerve surgery may reduce opioid use and nonopioid medication.^12^ There is also a high risk of use of psychotropic medication as well as psychoactive analgesics in nerve entrapment disorders.^4,5^ Here, we report the use of opioids and other specific drugs, such as gabapentinoids, amitriptyline, duloxetine, and venlafaxine, used to treat pain related to upper and lower limb nerve injuries in relation to surgery, together with demographic and socioeconomic status.

## 2. Methods

### 2.1. Dataset and individuals

Use of the defined pain medications at an individual level was analysed in a large Swedish record-linkage database, including medical/surgical diagnoses, and demographic and socioeconomic data as previously reported.^4,5^ The Total Swedish Population and the Longitudinal Integration Database for Health Insurance and Labour Market Studies (LISA), registers from Statistics Sweden (www.scb.se/en/) (these registers provide demographic and socioeconomic information), were linked to 3 registers administered by the National Board of Health and Welfare (www.socialstyrelsen.se/en/), ie, (1) the National Patient Register (which includes discharge diagnoses from hospital and outpatient clinics according to International Classification of Diseases and Causes of Death, 10th version [ICD-10] as well as diagnostic and surgical procedures according to the Swedish version of the NOMESCO Classification of Surgical Procedures [NCSP]), (2) the Cause of Death Register, and (3) the Swedish Prescribed Drug Register (which includes drug dispensations in Swedish pharmacies, but not stockpiles in nursing homes and hospital wards; coded according to the Anatomical Therapeutic Chemical [ATC] classification system).^4^ Unique personal identification numbers were used for linkage.

**Figure 1.**
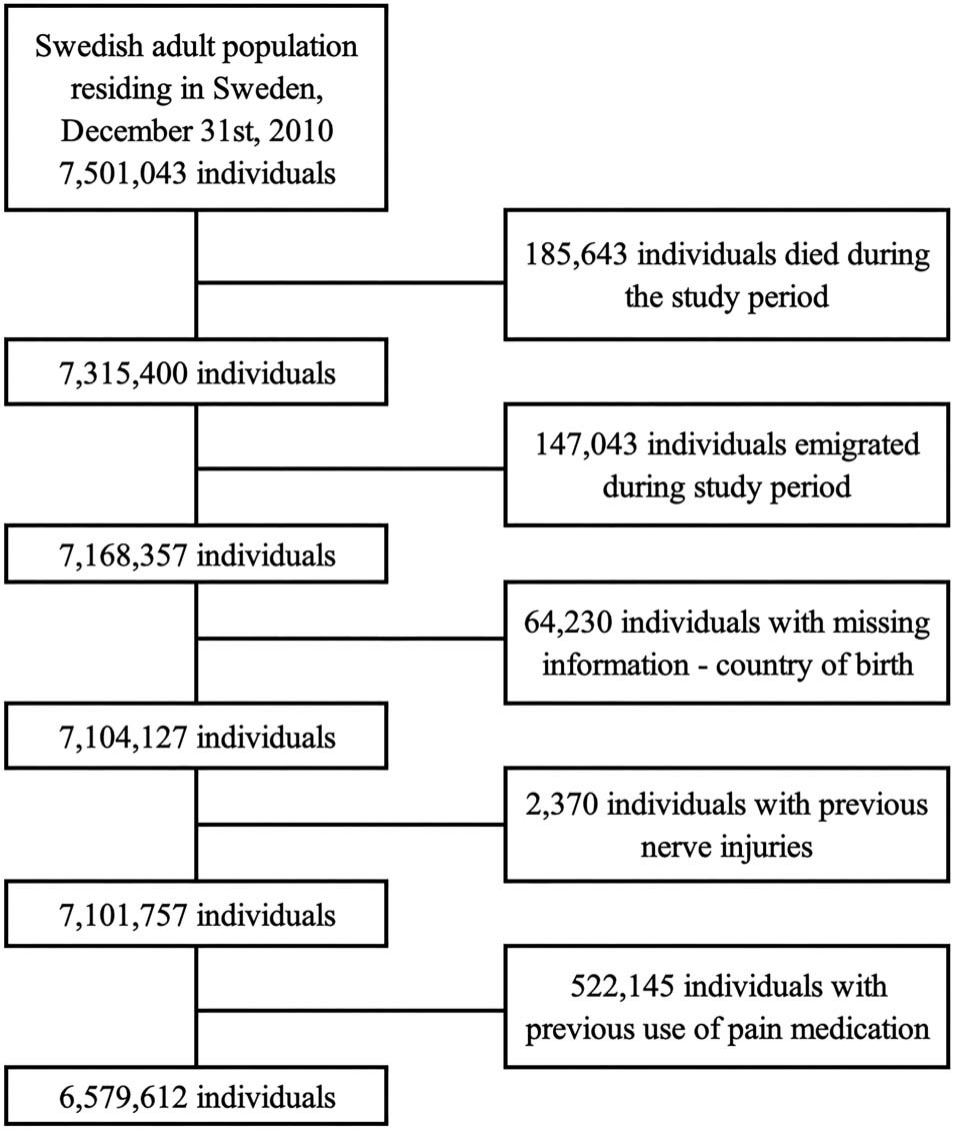
Flowchart showing the present population and the patient-based samples. The populations used to analyse the relation between having a nonsurgically and surgically treated nerve injury in the upper or lower limb and the consumption of pain medication.

We identified individuals, aged ≥18 years, residing in Sweden on December 31, 2010 (n = 7,501,043), after exclusion (Fig. 1). The exclusion criteria were: (1) death; (2) emigration during study period; (3) missing information concerning country of birth; (4) previous nerve injury (similar as present injuries); and (5) previous pain medication consumption. The final population comprised 6,579,612 individuals living between 2006 and 2014. Associations were estimated as relative risk (RR), 95% confidence interval (CI) obtained from logistic regression models (model 1 unadjusted and model 2 adjusted).

### 2.2. Assessment of variables

**Figure 2.**
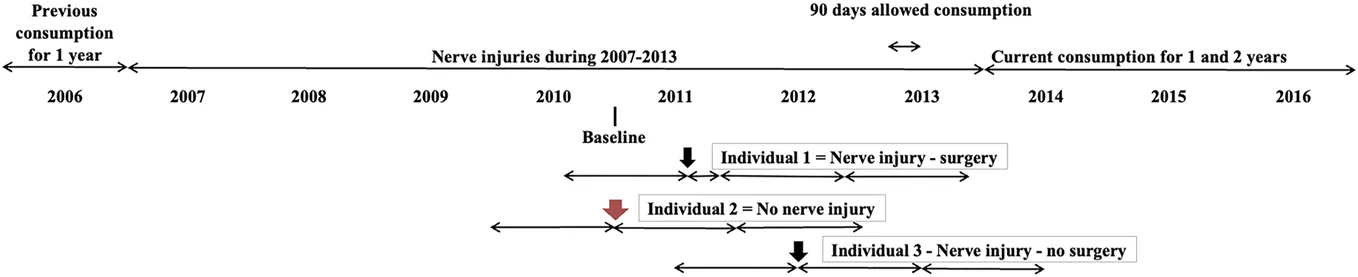
Schematic drawing of the principles of the consumption of pain medication in individuals without a nerve injury (individual 2) and in individuals with surgically (individual 1) and nonsurgically (individual 3) treated nerve injuries in the upper and lower limbs. Individuals without any nerve injury were sampled from December 31, 2010, whereas individuals with a nonsurgically or a surgically treated nerve injury in the upper or lower limbs, according to the definition (Supplemental Table 1, http://links.lww.com/PR9/A372), were sampled during the study period 2007 to 2013, where the cases were followed for 1 or 2 years. In the figure, examples are provided of individuals without and with a nerve injury. The first individual (no. 1; surgically treated) received the nerve injury diagnosis on July 31, 2011, the subsequent follow-up for consumption of pain medication, with a washout-period of 90 days, is indicated to the points of November 30, 2012, and November 30, 2013. For the second individual (no. 2), without a nerve injury diagnosis between 2011 and 2012, any nerve injury during 2010 was assessed and followed for consumption of pain medication in 2011 and 2012. The third individual (no. 3; nonsurgically treated) received the diagnosis on July 1, 2012, and the consumption of pain medication was assessed from that date, without a washout period, over a year to June 30, 2013, and then during the second year to June 30, 2014.

We identified individuals with nonsurgically and surgically treated nerve injuries according to defined ICD-10 and NCSP codes (Supplemental file 1, http://links.lww.com/PR9/A372). Most patients with a first surgery episode during the study period, 2007 to 2013, had a specific code for only one nerve injury in the upper or lower limb. Among these, wrist and hand (ICD-code S64) and calf (ICD-code S84) nerve injuries were most frequently operated on. For individuals who had their first surgery episode within the study period, we searched for any episodes within the previous year. If such an episode existed and had a similar code during the study period, the episode was excluded because our intention was to identify only first-ever surgery episodes during the study period (Fig. 2).

#### 2.2.1. Outcome variables

The dispensations of the analysed drugs, ie, opioids, gabapentinoids and 3 drugs used to treat neuropathic pain according to national guidelines (tricyclic antidepressants, and serotonin-norepinephrine reuptake inhibitors [SNRIs]), were defined based on their specific ATC codes: N02A (opioids), R05DA04 (codeine), N03AX16 (pregabalin), N03AX12 (gabapentin), N06AA09 (amitriptyline), N06AX21 (duloxetine), and N06AX16 (venlafaxine). For simplicity, we refer to the analyzed drugs collectively as “pain medications.” We considered pain medication use for 1 and 2 years. We allowed a washout period of 90 days only from *surgery* episodes (in sensitivity analysis in the Discussion, a 90-day washout period was applied to *all* individuals). Inclusion of any appropriate use of the defined drugs during the immediate postoperative period was thereby avoided. December 31, 2010, was used as the index date for individuals *with no* nonsurgically or surgically treated nerve injuries in upper or lower limbs. For these control cases, with no nerve injury episodes, that date was used as the start of follow-up and the end of the prior period of pain medication (Fig. 2).

Outcome was defined in 2 forms: (1) Time covered by drugs for 1 year was calculated using the cumulative number of defined daily doses (DDDs) of dispensations divided by 365. We arbitrarily defined a binary variable of pain medication use after diagnosis or diagnosis and surgery as “1” if the proportion of time covered within the evaluated year was >60% or otherwise as “0”. (2) In a multinominal analysis, pain medication use was categorized as (i) no prescription, (ii) 1 to 3, and (iii) >3 prescriptions during the year. When adjusting in the second year, previous pain medication use refers to consumption during the first year. We did not adjust for consumption in the first year because it is included in the exclusion criteria. Thus, individuals with no prior use of pain medication entered the cohort.

Other demographic variables were included and defined as age (5 categories 18–34 = reference, 34–44, 45–54, 65–79, and ≥80 years), sex (man = reference or woman), previous pain medication use during the first year adjusted in the second year (no = reference and yes), country of birth (native; ie, born in Sweden = reference or not; ie, immigrant), individualised disposable family income (low, middle, or high = reference income; as described),^4,5^ and cohabiting (yes = reference or not according to LISA database).^4,5^

### 2.3. Statistical analyses

First, the demographic and socioeconomic characteristics of the population with and without upper and lower limb nerve injuries, as well as use of pain medications after diagnosis (nonsurgically treated) or diagnosis and surgery (surgically treated) were described (Table 1). Two different logistic regression models were then applied to evaluate pain medication use: unadjusted in model 1, adjusted in model 2 for age, sex, previous use of pain medication during first year follow-up (ie, covariate in second year analysis), income, country of birth, and cohabiting (Table 2). Because the outcome prevalence was relatively low in several categories, odds ratios from the logistic regression models were interpreted as approximate RRs with 95% CIs, reflecting associations between explanatory variables and pain medication use. The multinomial regressions are presented as relative risks (RRs, 95% CI) for having 1 to 3 prescriptions or >3 prescriptions for pain medications, compared with no prescriptions (reference category), during the first and second years of follow-up.

**Table 1:** Demographic and socioeconomic data of a Swedish population with and without surgically treated nerve injuries in the upper and lower limbs.

	Nerve injuries		
	No	Lower limb	Upper limb
	6,569,096 (99.84)	1,274 (0.02)	9,242 (0.14)
Consumption of pain medications first year			
No	6,295,558 (95.84)	1,042 (81.79)	8,201 (88.74)
Yes	273,538 (4.16)	232 (18.21)	1,041 (11.26)
Consumption of pain medication second year			
No	6,239,877 (94.99)	1,105 (86.73)	8,459 (91.53)
Yes	329,219 (5.01)	169 (13.27)	783 (8.47)
Surgically treated			
No	6,569,096 (100)	1,260 (98.90)	5,249 (59.08)
Yes	—	14 (1.10)	3,669 (39.70)
Sex			
Men	3,288,624 (50.06)	684 (53.69)	6,211 (67.20)
Women	3,280,471 (49.94)	590 (46.31)	3,031 (32.80)
Age (y)			
18–34	1,876,126 (28.56)	421 (33.05)	3,465 (37.49)
34–44	1,166,843 (17.76)	220 (17.27)	1,767 (19.12)
45–54	1,096,883 (16.70)	204 (16.01)	1,598 (17.29)
55–64	1,048,305 (15.96)	201 (15.78)	1,250 (13.53)
65–79	1,051,629 (16.01)	168 (13.19)	956 (10.34)
≥80	329,310 (5.01)	60 (4.71)	206 (2.23)
Income			
Low	1,887,554 (28.73)	378 (29.67)	2,966 (32.09)
Middle	2,279,015 (34.69)	442 (34.69)	3,243 (35.09)
High	2,402,527 (36.57)	454 (35.64)	3,033 (32.82)
Country of birth			
Immigrant	980,508 (14.93)	181 (14.21)	1,205 (13.04)
Native	5,588,588 (85.07)	1,093 (85.79)	8,037 (86.96)
Cohabiting			
Living alone	3,157,089 (48.06)	693 (54.40)	5,080 (54.97)
Cohabiting	3,412,007 (51.94)	581 (45.60)	4,162 (45.03)

Values are n (%). Surgeries were only calculated for the exposure group (“no cases” present 0 surgically treated). For definition of opioids and drugs to treat neuropathic pain (“pain medication”), see Methods. Pharmacological drugs, defined as “pain medication”: 59% opioids and 41% pharmacological substances used in Sweden to counter neuropathic pain (see Results for details).

**Table 2:** Risk for use of opioids and pharmacological drugs to treat neuropathic pain (pain medication, see Methods), expressed as a binary variable (1 = 60% of the year covered), in a Swedish population with and without surgically treated (postsurgical washout period 90 days) nerve injuries in the upper and lower limbs.

		First year		Second year	
		Model 1	Model 2	Model 1	Model 2
Nerve injuries	No	Reference	Reference	Reference	Reference
	Lower limb	5.12 (4.44–5.91)	5.60 (4.85–6.48)	1.64 (1.36–1.96)	1.83 (1.52–2.19)
	Upper limb	4.20 (3.91–4.52)	4.85 (4.51–5.23)	1.19 (1.08–1.32)	1.41 (1.28–1.56)
Surgically treated	No	Reference	Reference	Reference	Reference
	Yes	0.28 (0.24–0.33)	0.31 (0.26–0.36)	1.11 (0.94–1.31)	1.16 (0.98–1.37)
Previous consumption of pain medications	No			Reference	Reference
	Yes			13.31 (13.20–13.43)	11.45 (11.35–11.56)
Sex	Men		Reference		Reference
	Women		1.25 (1.24–1.26)		1.22 (1.21–1.23)
Age (y)	18–34		Reference		Reference
	34–44		1.59 (1.56–1.61)		1.44 (1.42–1.46)
	45–54		2.02 (2.00–2.05)		1.78 (1.76–1.81)
	55–64		2.58 (2.54–2.61)		2.15 (2.12–2.18)
	65–79		3.32 (3.27–3.36)		2.79 (2.76–2.83)
	≥80		4.50 (4.43–4.57)		3.93 (3.87–3.98)
Income	Low		1.30 (1.28–1.31)		1.30 (1.28–1.31)
	Middle		1.20 (1.19–1.21)		1.18 (1.17–1.20)
	High		Reference		Reference
Country of birth	Immigrant		1.09 (1.08–1.10)		1.03 (1.02–1.04)
	Native		Reference		Reference
Cohabiting	Living alone		1.11 (1.10–1.11)		1.10 (1.09–1.11)
	Cohabiting		Reference		Reference

Model 1 is unadjusted and model 2 is adjusted for demographic socioeconomic factors and shown as relative risks ratio (RR) with 95% confidence interval.

The discriminatory accuracy for each model, and their ability to distinguish between individuals who experience (“cases”) and those who do not (control) experience outcome, was calculated by calculating the area under the receiver operating characteristic curve (AUC; ie, ability to discriminate between positive and negative cases) in accordance with a previous publication^4,5^ (criteria by Hosmer and Lemeshow used for interpretation: absent or very weak discrimination [“random guess] = AUC = 0.5 to 0.6; poor discrimination = AUC >0.6 to ≤0.7; acceptable discrimination = AUC >0.7 to ≤0.8; excellent discrimination = AUC >0.8–0.90; outstanding discrimination = AUC > 0.90). Stata v14.1 (StataCorp, College Station, TX) was used for the analyses.

### 2.4. Ethics declarations

Patient consent was waived as anonymized data were used, obtained from Swedish national registers, as approved by the National Ethical Committee (#: 2014-856). The National Board of Health and Welfare and Statistics Sweden facilitated linkage procedure of the database, after revision and consent from the registers' own data safety committees, as well as the initial approval by the Regional Ethical Committee in South Sweden (#: 2014-856). The data were anonymized before Swedish authorities delivered the data. The research was performed in accordance with the Declaration of Helsinki.

## 3. Results

### 3.1. Demographic and socioeconomic data

A larger number (n = 9,242) of nerve injuries was found in the upper limb than in the lower limb (n = 1,274; 0.14% and 0.02%, respectively, of the total population; Table 1). Around 40% of upper limb nerve injuries were surgically treated compared with 1.14% in the lower limb. Among individuals with lower limb nerve injuries, around 54% were men, which was lower than in the upper limb (around 67%; Table 1). In individuals with upper limb nerve injuries, these were more frequently observed in lower age categories (up to 44 years). Individuals with upper limb nerve injuries occurred slightly more frequently in the low-income category and among those living alone (Table 1). Finally, pain medication use was more common among individuals with upper (11.26%) and lower (18.21%) limb nerve injuries during the first year. These numbers were lower during the second year (8.47% and 13.27%, respectively) (Table 1). Corresponding percentages in the healthy population were 4.16% and 5.01%, respectively (Table 1).

The prescriptions for the pain medications were distributed as follows: 59% opioids (N02AA01 = morphine 14%; N02AA05 = oxycodone 25%; N02AE01 = buprenorphine 8%; N02AX = other opioids, eg, tramadol 47%, and “others” [N02AA55; N02AG02; N02AB03; N02AJ; R05DA04 = 6%]), and 41% pharmacological substances used in Sweden against neuropathic pain (N06AA09 = amitriptyline 21%; N06AX16 = venlafaxine 25%; N06AX21 = duloxetine 13%; N03AX16 = pregabalin 27%; N03AX12 = gabapentin 14%).

### 3.2. Use of pain medications during the first year

Compared with the general population, RRs (95% CI; model 2; adjusted for surgery, previous consumption, and demographic and socioeconomic data) of pain medication use during the first year after diagnosis or diagnosis and surgery were higher among individuals with upper (4.85 [4.51–5.23]) and lower limb (5.60 [4.85–6.48]) nerve injuries. Those with surgically treated nerve injuries had a lower risk of pain medication use (0.31 [0.26–0.36]) than those with no surgery during the first year, although both surgical and non-surgical injury groups had substantially higher risks than the general population (Table 2). Higher RRs were observed among women, those with low and middle incomes, immigrants, and those living alone. Relative risks increased with age (Table 2). Variance Inflation Factors (VIFs) were calculated for all predictor variables (mean VIF = 1.41, individual VIFs ≤ 1.87), indicating absence of significant multicollinearity.

### 3.3. Use of pain medications during the second year

Relative risks (model 2; adjusted) during the second year after diagnosis, with or without surgery, were lower than during the first year; still with slightly higher RRs for the lower limb (1.83 [1.52–2.19]) than the upper limb (1.41 [1.28–1.56]) (Table 2). There was no risk of pain medication use if the injuries were surgically treated (1.16 [0.98–1.37]), but there was a high risk if there had been use during the first year (11.45 [11.35–11.56]) (Table 2). Relative risks for pain medication use increased with age and were higher in women (1.22 [1.21–1.23]) and in individuals living alone (1.10 [1.09–1.11]) compared with those who were cohabiting. Relative risk was also higher in individuals with low (1.30 [1.28–1.31]) and middle (1.18 [1.17–1.20]) incomes. Compared with natives, immigrants also presented a slightly higher risk of pain medication use during the second year (1.03 [1.02–1.04]) (Table 2).

Thus, during the second year compared with the first year, RRs for pain medication use diminished for both upper and lower limb nerve injuries, remained high among women, immigrants, those living alone, and among individuals with low and middle incomes, and increased with age. Use in the first year substantially increased the risk of use in the second year.

### 3.4. Number of prescriptions for pain medications during the first and second years

**Figure 3.**
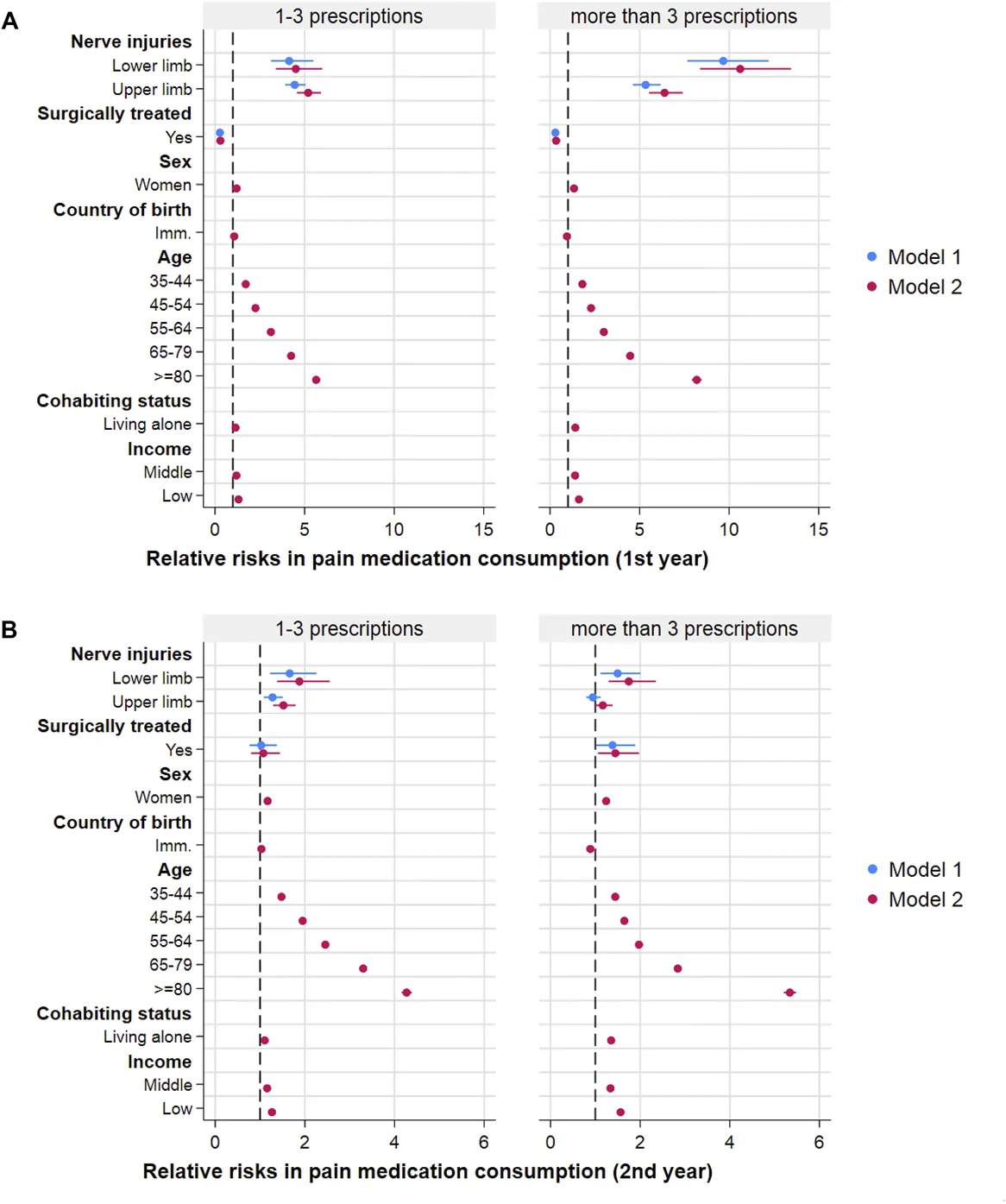
Relative risk for consumption of pain medication in nonsurgically and surgically treated upper and lower nerve injuries. Visualisation of relative risk (RR; with 95% confidence intervals) of consumption (reference no prescriptions and presented as 1–3 and >3 prescriptions) of pain medication (multinominal variable) in a Swedish population with and without surgically treated nerve injuries in the upper and lower limb during the first (A) and the second (B) years after diagnosis or diagnosis and surgery. Model 1 is unadjusted and model 2 is adjusted for demographic socioeconomic factors (including consumption during the first year; Table 3) and shown as RRs with 95% confidence interval.

Relative risks (95% CIs) are presented (Table 3; Fig. 3), based on the multinominal analysis, expressed as 1 to 3 and >3 prescriptions for pain medication according to the given definition (see Methods), compared with the reference “no prescriptions,” for 2 years. Relative risk patterns were essentially similar to the RRs with logistic regression with the binary variable, where the risk for 1 to 3 prescriptions of the drugs was high for both upper (5.20 [4.57–5.91]) and lower (4.51 [3.40–5.99]) limb nerve injuries (adjusted), and lower among individuals treated surgically than among those not treated (0.30 [0.22–0.40]) during the first year. Higher risks were observed among female sex, higher age, low and middle incomes, immigrants, and those living alone (Table 3; Fig. 3A).

**Table 3:** Risk for use of pain medication, expressed as no prescriptions of opioids or other pharmacological drugs to treat neuropathic pain (pain medication; see Methods) and 1–3 or >3 prescriptions (postsurgical washout period of 90 days) in a multinominal regression, in a Swedish population with and without surgically treated nerve injuries in the upper and lower limb.

Outcome			First year		Second year	
			Model 1	Model 2	Model 1	Model 2
No consumption of the defined drugs			Reference	Reference	Reference	Reference
Low, moderate consumption (1–3 prescriptions)	Nerve injury	No	Reference	Reference	Reference	Reference
		Lower limb	4.14 (3.12–5.48)	4.51 (3.40–5.99)	1.66 (1.22–2.26)	1.88 (1.38–2.55)
		Upper limb	4.45 (3.92–5.05)	5.20 (4.57–5.91)	1.28 (1.08–1.50)	1.52 (1.29–1.79)
	Previous consumption pain medication	No			Reference	Reference
		Yes			12.08 (11.89–12.26)	10.29 (10.13–10.45)
	Surgically treated	No		Reference		Reference
		Yes	0.27 (0.20–0.37)	0.30 (0.22–0.40)	1.03 (0.76–1.38)	1.07 (0.80–1.44)
	Sex	Men		Reference		Reference
		Women		1.20 (1.18–1.22)		1.17 (1.15–1.19)
	Age (y)	18–34		Reference		Reference
		34–44		1.71 (1.66–1.76)		1.48 (1.44–1.52)
		45–54		2.26 (2.20–2.32)		1.95 (1.90–2.00)
		55–64		3.12 (3.04–3.21)		2.46 (2.40–2.52)
		65–79		4.25 (4.14–4.36)		3.30 (3.23–3.38)
		≥80		5.64 (5.48–5.81)		4.27 (4.16–4.38)
	Income	Low		1.31 (1.29–1.34)		1.27 (1.24–1.29)
		Middle		1.19 (1.17–1.21)		1.16 (1.14–1.18)
		High		Reference		Reference
	Country of birth	Immigrant		1.07 (1.05–1.09)		1.03 (1.01–1.05)
		Native		Reference		Reference
	Cohabiting	Living alone		1.14 (1.13–1.16)		1.10 (1.09–1.12)
		Cohabiting		Reference		Reference
High consumption (>3 prescriptions)	Nerve injury	No	Reference	Reference	Reference	Reference
		Lower limb	9.67 (7.66–12.21)	10.62 (8.38–13.46)	1.50 (1.12–2.01)	1.75 (1.30–2.35)
		Upper limb	5.34 (4.62–6.17)	6.39 (5.52–7.40)	0.94 (0.80–1.12)	1.17 (0.98–1.38)
	Previous consumption of pain medication	No			Reference	Reference
		Yes			35.42 (34.94–35.93)	29.81 (29.38–30.25)
	Surgically treated	No		Reference		Reference
		Yes	0.29 (0.21–0.41)	0.34 (0.24–0.47)	1.38 (1.01–1.89)	1.45 (1.06–1.98)
	Sex	Men		Reference		Reference
		Women		1.34 (1.31–1.36)		1.24 (1.22–1.26)
	Age (y)	18–34		Reference		Reference
		34–44		1.80 (1.74–1.87)		1.44 (1.40–1.48)
		45–54		2.29 (2.21–2.37)		1.65 (1.60–1.69)
		55–64		3.00 (2.90–3.11)		1.97 (1.92–2.03)
		65–79		4.47 (4.34–4.62)		2.84 (2.77–2.91)
		≥80		8.19 (793–8.46)		5.34 (5.20–5.48)
	Income	Low		1.61 (1.57–1.65)		1.56 (1.53–1.59)
		Middle		1.40 (1.37–1.43)		1.34 (1.31–1.36)
		High		Reference		Reference
	Country of birth	Immigrant		0.94 (0.92–0.97)		0.89 (0.87–0.91)
		Native		Reference		Reference
	Cohabiting	Living alone		1.41 (1.38–1.43)		1.35 (1.33–1.37)
		Cohabiting		Reference		Reference

Model 1 is unadjusted and model 2 is adjusted for demographic socioeconomic factors and shown as relative risk (RR) with 95% confidence interval.

The risk of having >3 prescriptions was even higher among those individuals with upper (6.39 [5.52–7.40]) and lower (10.62 [8.38–13.46]) limb nerve injuries (adjusted); again, a lower risk was seen among surgically treated individuals compared with those without surgery (0.34 [0.24–0.47]) during the first year. Relative risks were increased for having >3 prescriptions during the first year, with a slight trend towards higher figures for demographic and socioeconomic factors, with the exception of being an immigrant (Table 3; Fig. 3A).

During the second year, there was a substantial decrease in RRs concerning both upper and lower limb nerve injuries for individuals with >3 prescriptions, down to the same levels as for individuals with 1 to 3 prescriptions, among whom a reduced risk was also observed (Table 3; Fig. 3B). One difference was found concerning RR for those with nerve surgery, ie, no risk of having 1 to 3 prescriptions but a higher risk of having >3 prescriptions (Table 3; Fig. 3B). The increased risk remained for women, increasing age, low and middle incomes, immigrants (lower, however, among those with >3 prescriptions), and living alone regarding 1 to 3 and >3 prescriptions (Table 3; Fig. 3B). A prominent feature was the increased risk of consistent consumption, particularly concerning >3 prescriptions (29.81 [29.38–30.25]) compared with a risk of having 1 to 3 prescriptions (10.29 [10.13–10.45]) in the second year.

## 4. Discussion

This nationwide study demonstrates a significantly increased risk of pain medication use among individuals with upper and lower limb nerve injuries, particularly in the first year postdiagnosis or -surgery. Notably, surgery was associated with a reduced risk of medication use during this period. The demographic and socioeconomic factors, which generally increased the risk, only marginally, included being a woman, being older, having low and middle income levels, being an immigrant and living alone. The currently used 90-day washout period post-surgery revealed a high and persisting risk of use, indicating that today's tapering and education strategies may be insufficient. In accordance, among individuals with a similar nerve injury, ie, surgically treated for neuroma, one third used pain medication >4 weeks (OR 4.41 [1.36–14.3]).^25^ A higher RR for overuse of opioids and gabapentinoid drugs is associated with surgery for nerve entrapment disorders, particularly UNE, when a population is divided according to such use occurring (1) before surgery only, (2) after surgery only, or (3) before and after surgery.^5^ The effect of introducing a standardized postoperative opioid prescription protocol after UNE surgery is probably explained by increased protocol adherence and greater vigilance among prescribers.^15^ A strategy for restricted, conservative, standardized, and safe pain medication prescription is urgently required^38^ because a preoperative misuse of pain medication is associated with morbidity and mortality.^30^

Interestingly, surgery, compared with no surgery, for nerve injuries reduced the risk for consumption during the first but not during the second year. Consumption was high, however, in individuals with upper and lower limb nerve injuries, with indications that the risk was higher among those affected in the lower limb. Upper and lower limb nerve surgery reduces opioid use, but, in partial agreement with our study of persistent use in the second year if used the first year, preoperative use of pain medication is associated with prolonged use.^25^ Lower limb nerve injuries probably have residual problems to a similar extent, affected by the type of nerve trunk involved, severity of nerve injury, mechanism(s) of injury, location, and affected branches of the nerve injury along the limb, and detailed type(s) of surgical procedure.^1,28,34^ These aspects cannot be sufficiently reflected using available ICD-10 and surgical codes. We included many upper and lower nerve injuries with adjustment for demographic and socioeconomic factors, which is a further aspect that affects the outcome of nerve injury and surgery. In addition, we compared pain medication use with a national population, allowing appropriate statistical analysis with sufficient power.

The observed persistent risk of pain medication consumption indicated that opioids, particularly oxycodone and tramadol (75%), dominated the prescription patterns. The risk of addiction and an insufficient pain response with opioid use over time is well known.^3,29^ Such treatment should be avoided or kept to a minimum in both dose and duration. Alterations in μ-opioid receptor coupling^26^ and opioid receptors in dorsal root ganglia neurons^37,41^ may be induced by a nerve injury. At the time of evaluation, tramadol was commonly used in Sweden for pain treatment, but has since decreased.^2^ However, globally, it remains a currently used drug.^22^ Intriguingly, recommended drugs in Sweden for neuropathic pain treatment (eg, gabapentinoids, tricyclic antidepressants, and SNRIs) showed a distribution frequency of 13% to 27%. Such drugs are first-line options for pain treatment in nerve injuries. Opioids, however, while accounting for most prescriptions (59%), are third-line options for treating neuropathic pain. Yet, opioid use exceeded that of the recommended first-line treatments in both Swedish and international guidelines. Thus, our data suggest that prescribing practices do not align with recommendations and may reflect inappropriate prescribing. The prescribing behavior of physicians, with possible impacts from national guidelines and specific demands made by the affected individual, should be considered. These are factors that certainly interact.^20^

Nerve surgery during the first year was associated with a lower risk for both low and high consumption of the drugs, indicating the relevance of appropriate nerve surgery if possible. Nerve surgery may reduce the use of opioid and nonopioid medications.^12^ Surgery for digital nerve injuries has been questioned because complete functional recovery may not be achieved.^8,21^ However, pain problems should also be considered in such injuries. The present findings, along with other published data on neuroma treatment, indicate that surgical treatment reduces opioid use during the first year with no higher risk over time.^7,25^ Notably, if an individual consumes painkillers during the first year, there is a substantially increased risk of particularly high consumption during the second year. This is in agreement with data from individuals with surgically treated neuroma, who have a higher risk for persistent opioid use >4 weeks after surgery if treated with opioids preoperatively.^25^ A similar trend is seen in brachial plexus injuries.^9^

Persistent pain, defined as neuropathic pain in 73% of instances, is reported in around half of individuals with a repaired or reconstructed upper limb nerve injury.^32^ Sex differences in pain sensitivity are also described after nerve injuries.^33^ Demographic and socioeconomic factors, including older age, female sex, immigrant status, low/middle income, and living alone, were independently associated with increased drug use. These findings echo patterns seen in nerve entrapment disorders^4,5^ and underline the social determinants of pain management. Interindividual differences may be even more relevant than age and sex^16^ and a concomitant mental disorder may also influence the risk of opioid consumption.^40,43^

The elevated risk of prolonged use of pain medication presented here reflects broader international concerns around opioid overprescription and its consequences. Guidelines for opioid prescribing, published by the Center for Disease Control and Prevention, emphasize the avoidance of routine opioid use for chronic noncancer pain, preferring nonopioid therapies, and careful evaluation of risk-benefit profiles before initiating treatment. Similarly, the World Health Organization recommends structured opioid stewardship programs that include prescriber education, patient risk stratification, and multidisciplinary approaches to pain management. In light of these frameworks, our findings support the integration of preoperative pain medication risk screening, particularly in socioeconomically vulnerable groups, and underline the potential for early surgery to reduce dependency. The persistence of high-risk use among certain demographic groups calls for the development of multidisciplinary postoperative care models, incorporating psychological support, physiotherapy, and socioeconomic assessment to improve outcomes and ensure alignment with global opioid stewardship standards. Despite international differences in health care systems, our data provide information about the risks posed by the use of pain medication, when opioids, particularly tramadol, are prescribed for upper and lower limb nerve injuries. Tramadol and other opioids, despite being a non-recommendable drug, continue to be prescribed globally with wide differences in praxis among countries.^22,35,39^ Thus, our data provide the possibility of generalizability regarding pain medication in nerve injuries.

There are limitations in this current work. First, we cannot evaluate the intent of the prescriber or the appropriateness of the prescription because we cannot adjust for nerve injury severity, location and nature of the nerve injury or the extent of the surgeries, and a number of unknown clinical indicators, including individual pain vulnerability, that may affect the prescribing pattern, but we were able to integrate socioeconomic factors. Another limitation is that we excluded those individuals with a previous use of pain medication, which may affect both generalizability and risk of the data not being applicable to a “real-world” population. The definition of pain medication use can be discussed. We used a definition previously applied, namely as the cumulative number of DDDs of dispensations divided by 365, with a defined proportion of >60% of covered time as long-term use during 1 or 2 years, which we considered as valid.^5^ Other published definitions are based on number of prescriptions, number of days' supply, oral equivalent units, or continuous episodes of long-term pain medication therapy.^24^

We conducted a sensitivity analysis to assess the potential impact of applying the 90-day washout period, which in the main analysis was considered only for individuals undergoing surgery. When this definition was extended to all individuals (ie, controls, diagnosed individuals without surgery, and diagnosed individuals with surgery), we observed RRs (95% CI) of pain medication use in the first year of 2.67 (2.21–3.23) for lower limb and 2.02 (1.83–2.23) for upper limb, whereas RR for surgery was 0.61 (0.51–0.73). In the second year, RRs decreased to 1.89 (1.55–2.31) for lower limb and 1.52 (1.37–1.69) for upper limb, with RR for surgery increasing to 0.89 (0.75–1.06). These findings are consistent with our main results, although RRs were attenuated when applying the 90-day washout period across all groups.

A last limitation is that we cannot, because of the study design (observational study), draw any conclusion about causality,^19^ but we observed associations between nonsurgically and surgically treated nerve injuries in upper and lower limbs, demographic and socioeconomic status, and pain medication use. We cannot exclude the possibility that the associations observed are influenced by some unmeasured confounders, such as health literacy, comorbidities, and mental health or behavior, for example, seeking or using the health care system. Our study is based on data from the Swedish health care system, which is tax-funded and provides universal coverage, with strict regulation and monitoring of prescribed drugs through nationwide registers. This implies a potential impact on comparisons with other national health care systems. Nevertheless, underlying biological risks of neuropathic pain after nerve injury, and socioeconomic disparities in medication use, are likely to be relevant across diverse settings.

## 5. Conclusions

In conclusion, from a population-level perspective, individuals with nerve injuries face an increased risk of long-term pain medication use. Early surgery seems to mitigate short-term dependency, but demographic and socioeconomic disparities remain risk factors. These findings call for targeted perioperative strategies, equitable access to surgery, and robust pain medication stewardship programs.

## Disclosures

The authors have no conflict of interest to declare.

## Supplemental digital content

Supplemental digital content associated with this article can be found online at http://links.lww.com/PR9/A372.

## Supplementary Material

## References

[1] Asplund M Nilsson M Jacobsson A von Holst H. Incidence of traumatic peripheral nerve injuries and amputations in Sweden between 1998 and 2006. Neuroepidemiology 2009;32:217–28. 10.1159/000197900 19174611

[2] Bardage C Grünewald M Tuvendal P Ljung R. First opioid prescribing in Sweden: drugs, doses, and diagnoses in more than 600 000 opioid-naïve and cancer free patients. J Substance Use 2023;29:617–23.

[3] Chou R Turner JA Devine EB Hansen RN Sullivan SD Blazina I Dana T Bougatsos C Deyo RA. The effectiveness and risks of long-term opioid therapy for chronic pain: a systematic review for a National Institutes of Health Pathways to Prevention Workshop. Ann Int Med 2015;162:276–86. 10.7326/M14-2559 25581257

[4] Dahlin LB Perez R Nyman E Zimmerman M Merlo J. Carpal tunnel syndrome and ulnar nerve entrapment are associated with impaired psychological health in adults as appraised by their increased use of psychotropic medication. J Clin Med 2022;11:3871. 10.3390/jcm11133871 PMC9267822 35807165

[5] Dahlin LB Perez R Nyman E Zimmerman M Merlo J. Overuse of the psychoactive analgesics' opioids and gabapentinoid drugs in patients having surgery for nerve entrapment disorders. Sci Rep 2023;13:16248. 10.1038/s41598-023-43253-0 PMC10533484 37758760

[6] Dahlin LB Wiberg M. Nerve injuries of the upper extremity and hand. EFORT Open Rev 2017;2:158–70. 10.1302/2058-5241.2.160071 PMC5467675 28630754

[7] Ducic I Yoon J Eberlin KR. Treatment of neuroma-induced chronic pain and management of nerve defects with processed nerve allografts. Plast Reconstr Surg Glob Open 2019;7:e2467. 10.1097/GOX.0000000000002467 PMC7288900 32537284

[8] Dunlop RLE Wormald JCR Jain A. Outcome of surgical repair of adult digital nerve injury: a systematic review. BMJ Open 2019;9:e025443. 10.1136/bmjopen-2018-025443 PMC6429897 30872549

[9] Dy CJ Peacock K Olsen MA Ray WZ Brogan DM. Frequency and risk factors for prolonged opioid prescriptions after surgery for brachial plexus injury. J Hand Surg 2019;44:662–8.e1. 10.1016/j.jhsa.2019.04.001 PMC7193763 31078338

[10] Evertsson L Bjorkman A Turesson C Arner M Mellstrand Navarro C. Long-term subjective and objective outcomes after digital nerve repair: a cohort study. J Hand Surg Eur Vol 2025;50:649–58. 10.1177/17531934241286116 PMC12012279 39397395

[11] Faust A DeMartini SJ Carey-Ewend A Crock LW Buday SK Brogan DM Dy CJ. Concepts of pain management following nerve injuries: multidisciplinary approach. J Hand Surg Glob Online 2024;6:749–55. 10.1016/j.jhsg.2024.01.019 PMC11456641 39381396

[12] Felder JM Ducic I. Impact of nerve surgery on opioid and medication use in patients with chronic nerve injuries. Plast Reconstr Surg Glob Open 2021;9:e3789. 10.1097/GOX.0000000000003789 PMC8423382 34513538

[13] Frostadottir D Ekman L Zimmerman M Andersson S Arner M Brogren E Dahlin LB. Cold sensitivity, functional disability and predicting factors after a repaired digital nerve injury. Sci Rep 2022;12:4847. 10.1038/s41598-022-08926-2 PMC8941129 35318398

[14] Frostadottir D Ekman L Zimmerman M Dahlin LB. Cold sensitivity and its association to functional disability following a major nerve trunk injury in the upper extremity—a national registry-based study. PLoS One 2022;17:e0270059. 10.1371/journal.pone.0270059 PMC9275699 35819958

[15] Giberson-Chen C Liu C Grisdela P Jr Liu D Model Z Steele A Blazar P Earp BE Zhang D. Evaluating prescriber adherence to a standardized postoperative opioid prescription protocol for cubital tunnel surgery. J Hand Surg Glob Online 2024;6:374–81. 10.1016/j.jhsg.2024.02.007 PMC11133905 38817747

[16] Graeff P Itter A Wach K Ruscheweyh R. Inter-individual differences explain more variance in conditioned pain modulation than age, sex and conditioning stimulus intensity combined. Brain Sci 2021;11:1186. 10.3390/brainsci11091186 PMC8468738 34573207

[17] Gutierrez T Oliva I Crystal JD Hohmann AG. Peripheral nerve injury promotes morphine-seeking behavior in rats during extinction. Exp Neurol 2021;338:113601. 10.1016/j.expneurol.2021.113601 PMC8351527 33453217

[18] Higginbotham JA Markovic T Massaly N Moron JA. Endogenous opioid systems alterations in pain and opioid use disorder. Front Syst Neurosci 2022;16:1014768. 10.3389/fnsys.2022.1014768 PMC9628214 36341476

[19] Hill AB. The environment and disease: association or causation? Proc R Soc Med 1965;58:295–300. 10.1177/003591576505800503 PMC1898525 14283879

[20] Hjerpe P Ohlsson H Lindblad U Bostrom KB Merlo J. Understanding adherence to therapeutic guidelines: a multilevel analysis of statin prescription in the Skaraborg Primary Care Database. Eur J Clin Pharmacol 2011;67:415–23. 10.1007/s00228-010-0973-4 21190018

[21] Jain A Dunlop R Hems T Tang JB. Outcomes of surgical repair of a single digital nerve in adults. J Hand Surg Eur Vol 2019;44:560–5. 10.1177/1753193419846761 31079523

[22] Jayawardana S Forman R Johnston-Webber C Campbell A Berterame S de Joncheere C Aitken M Mossialos E. Global consumption of prescription opioid analgesics between 2009-2019: a country-level observational study. EClinicalMedicine 2021;42:101198. 10.1016/j.eclinm.2021.101198 PMC8599097 34820610

[23] Khouri AN Chung KC. Evaluating outcomes following nerve repair: beyond the medical research council. Hand Clin 2024;40:441–9. 10.1016/j.hcl.2024.03.005 38972688

[24] Kristiansen EB Pedersen AB. Variation in risk of opioid therapy and association with mortality following hip or knee arthroplasty: an analysis based on 14 different definitions. Acta Orthop 2025;96:664–70. 10.2340/17453674.2025.44572 PMC12404099 40891928

[25] Lans J Westenberg RF Gottlieb RE Valerio IL Chen NC Eberlin KR. Long-term opioid use following surgery for symptomatic neuroma. J Reconstr Microsurg 2022;38:137–43. 10.1055/s-0041-1731640 35100646

[26] Largent-Milnes TM Guo W Wang HY Burns LH Vanderah TW. Oxycodone plus ultra-low-dose naltrexone attenuates neuropathic pain and associated μ-opioid receptor–Gs coupling. J Pain 2008;9:700–13. 10.1016/j.jpain.2008.03.005 PMC5469510 18468954

[27] Magistroni E Parodi G Fop F Battiston B Dahlin LB. Cold intolerance and neuropathic pain after peripheral nerve injury in upper extremity. J Peripher Nervous Syst 2020;25:184–90. 10.1111/jns.12376 32297385

[28] Magneli M Axenhus M. Epidemiology and regional variance of traumatic peripheral nerve injuries in Sweden: a 15-year observational study. PLoS One 2024;19:e0310988. 10.1371/journal.pone.0310988 PMC11463750 39383132

[29] McNicol ED Midbari A Eisenberg E. Opioids for neuropathic pain. Cochrane Database Syst Rev 2013;2013:CD006146. 10.1002/14651858.CD006146.pub2 PMC6353125 23986501

[30] Menendez ME Ring D Bateman BT. Preoperative opioid misuse is associated with increased morbidity and mortality after elective orthopaedic surgery. Clin Orthop Relat Res 2015;473:2402–12. 10.1007/s11999-015-4173-5 PMC4457771 25694266

[31] Miclescu A Essemark M Astermark M Gkatziani P Straatmann A Butler S Karlsten R Gordh T. Prolonged time of after-sensation after experimental pain stimuli despite efficient conditioned pain modulation in patients with chronic neuropathic pain after traumatic nerve injuries in upper extremity. Pain Rep 2021;6:e908. 10.1097/PR9.0000000000000908 PMC7935643 33688603

[32] Miclescu A Straatmann A Gkatziani P Butler S Karlsten R Gordh T. Chronic neuropathic pain after traumatic peripheral nerve injuries in the upper extremity: prevalence, demographic and surgical determinants, impact on health and on pain medication. Scand J Pain 2019;20:95–108. 10.1515/sjpain-2019-0111 31536038

[33] Miclescu AA Gkatziani P Granlund P Butler S Gordh T. Sex-related differences in experimental pain sensitivity in subjects with painful or painless neuropathy after surgical repair of traumatic nerve injuries. Pain Rep 2022;7:e1033. 10.1097/PR9.0000000000001033 PMC9586924 36284797

[34] Murphy RNA de Schoulepnikoff C Chen JHC Columb MO Bedford J Wong JK Reid AJ. The incidence and management of peripheral nerve injury in England (2005-2020). J Plast Reconstr Aesthet Surg 2023;80:75–85. 10.1016/j.bjps.2023.02.017 36996504

[35] Nestvold HH Skurtveit SS Hamina A Hjellvik V Odsbu I. Socioeconomic risk factors for long-term opioid use: a national registry-linkage study. Eur J Pain 2024;28:95–104. 10.1002/ejp.2163 37501355

[36] Portincasa A Gozzo G Parisi D Annacontini L Campanale A Basso G Maiorella A. Microsurgical treatment of injury to peripheral nerves in upper and lower limbs: a critical review of the last 8 years. Microsurgery 2007;27:455–62. 10.1002/micr.20382 17596860

[37] Robertson B Schulte G Elde R Grant G. Effects of sciatic nerve injuries on *δ* ‐opioid receptor and substance P immunoreactivities in the superficial dorsal horn of the rat. Eur J Pain 1999;3:115–29. 10.1053/eujp.1998.0104 10700341

[38] Saloner B McGinty EE Beletsky L Bluthenthal R Beyrer C Botticelli M Sherman SG. A public health strategy for the opioid crisis. Public Health Rep 2018;133:24S–34S. 10.1177/0033354918793627 PMC6243441 30426871

[39] Samuelsen PJ Hjellvik V Odsbu I Stubhaug A Skurtveit S Vambheim SM. Trends in postoperative opioid use in Norway 2011-2018: a nationwide registry-based study. Basic Clin Pharmacol Toxicol 2025;136:e70040. 10.1111/bcpt.70040 40289303

[40] Santo T Jr Campbell G Gisev N Martino-Burke D Wilson J Colledge-Frisby S Clark B Tran LT Degenhardt L. Prevalence of mental disorders among people with opioid use disorder: a systematic review and meta-analysis. Drug Alcohol Depend 2022;238:109551. 10.1016/j.drugalcdep.2022.109551 35797876

[41] Sun L Zhao JY Gu X Liang L Wu S Mo K Feng J Guo W Zhang J Bekker A Zhao X Nestler EJ Tao YX. Nerve injury-induced epigenetic silencing of opioid receptors controlled by DNMT3a in primary afferent neurons. PAIN 2017;158:1153–65. 10.1097/j.pain.0000000000000894 PMC5435541 28267064

[42] Thorsen F Rosberg HE Steen Carlsson K Dahlin LB. Digital nerve injuries: epidemiology, results, costs, and impact on daily life. J Plast Surg Hand Surg 2012;46:184–90. 10.3109/2000656X.2012.676554 22686434

[43] Ward R Tang YL Axon RN Casarella J Whitfield N Rauch SAM. Effectiveness of a substance use treatment program for veterans with chronic pain and opioid use disorder. J Substance Abuse Treat 2022;132:108635. 10.1016/j.jsat.2021.108635 34607731

[44] Zhao C Tall JM Meyer RA Raja SN. Antiallodynic effects of systemic and intrathecal morphine in the spared nerve injury model of neuropathic pain in rats. Anesthesiology 2004;100:905–11. 10.1097/00000542-200404000-00021 15087626

