# Supplementary material for: Pain medication and nerve injuries in upper or lower limb—a Swedish national registry study
Source: Pain Rep. 2026 Jan 16;11(1):e1391. doi: 10.1097/PR9.0000000000001391 (PMC12815522; doi:10.1097/PR9.0000000000001391)
Supplement: SUPPLEMENTARY MATERIAL [file painreports-11-e1391-s001.pdf]

Supplemental file 1

ICD-10 codes for nerve injuries (upper limb and lower limb)

Upper limb:
S.44 Nerve injuries at shoulder and in upper arm

S.54 Nerve injuries in forearm

S.64 Nerve injuries at wrist and in hand

Lower limb:
S.74 Nerve injuries at hip and in thigh

S.84 Nerve injuries in calf

S.94 Nerve injuries at ankle and in foot

Surgical procedures related to nerve injuries

Upper limb:

ACB21	Suture of peripheral nerve – median nerve
ACB22	Suture of peripheral nerve – radial nerve
ACB23	Suture of peripheral nerve – ulnar nerve
ACB29	Suture of peripheral nerve – other or unspecified nerve
ACC21	Reconstruction of peripheral nerve - median nerve
ACC22	Reconstruction of peripheral nerve – radial nerve
ACC23	Reconstruction of peripheral nerve – ulnar nerve
ACC29	Reconstruction of peripheral nerve - other or unspecified nerve

Lower limb:

ACB24	Suture of peripheral nerve – peroneal nerve
ACB25	Suture of peripheral nerve – tibial nerve
ACB26	Suture of peripheral nerve – sciatic nerve
ACC24	Reconstruction of peripheral nerve – peroneal nerve
ACC25	Reconstruction of peripheral nerve – tibial nerve
ACC26	Reconstruction of peripheral nerve – sciatic nerve

ACB29 and ACC29 were classified as procedures in the upper limb
